# L-Theanine Improves the Gelation of Ginkgo Seed Proteins at Different pH Levels

**DOI:** 10.3390/gels10020131

**Published:** 2024-02-06

**Authors:** Luyan Zhang, Huifang Ge, Jing Zhao, Changqi Liu, Yaosong Wang

**Affiliations:** 1 Department of Food Science and Engineering, College of Light Industry and Food Engineering, Nanjing Forestry University, Nanjing 210037, China; luyanzhang@njfu.edu.cn; 2 State Key Laboratory of Tea Plant Biology and Utilization, Anhui Agricultural University, 130 Changjiang West Road, Hefei 230036, China; ghfjulia@ahau.edu.cn; 3 School of Exercise and Nutritional Sciences, San Diego State University, San Diego, CA 92182, USA; jzhao2@sdsu.edu (J.Z.); changqi.liu@sdsu.edu (C.L.)

**Keywords:** ginkgo seed protein isolate, L-theanine, gel strength, water holding capacity, microstructure

## Abstract

L-theanine (L-Th), a non-protein amino acid naturally found in teas and certain plant leaves, has garnered considerable attention due to its health benefits and potential to modify proteins such as ginkgo seed proteins, which have poor gelling properties, thereby expanding their applications in the food industry. The objective of this study was to investigate the impact of varying concentrations of L-Th (0.0%, 0.5%, 1.0%, and 2.0%) on the gelling properties of ginkgo seed protein isolate (GSPI) at various pH levels (5.0, 6.0, and 7.0). The GSPI gels exhibited the highest strength at a pH of 5.0 (132.1 ± 5.6 g), followed by a pH of 6.0 (95.9 ± 3.9 g), while a weak gel was formed at a pH of 7.0 (29.5 ± 0.2 g). The incorporation of L-Th increased the hardness (58.5–231.6%) and springiness (3.0–9.5%) of the GSPI gels at a pH of 7.0 in a concentration-dependent manner. However, L-Th did not enhance the gel strength or water holding capacity at a pH of 5.0. The rheological characteristics of the GSPI sols were found to be closely related to the textural properties of L-Th-incorporated gels. To understand the underlying mechanism of L-Th’s effects, the physicochemical properties of the sols were analyzed. Specifically, L-Th promoted GSPI solubilization (up to 7.3%), reduced their hydrophobicity (up to 16.2%), reduced the particle size (up to 40.9%), and increased the ζ potential (up to 21%) of the sols. Overall, our findings suggest that L-Th holds promise as a functional ingredient for improving gel products.

## 1. Introduction

The rapid growth of the world’s population, coupled with changing sociodemographic structures, is poised to exert enormous pressure on global resources to supply nutritious food [1]. Proteins, which are crucial for human growth and health, become a focal point when animal proteins fall short of meeting the demands of a growing population. Plant-based proteins emerged as a sustainable alternative due to their low environmental footprint and potential health benefits [2,3,4].

Extensively utilized in food processing, plant-based food proteins contribute significantly to the nutritional values and sensory attributes (e.g., texture, flavor, or color) of various food products [5,6]. For instance, proteins from cereals, pseudocereals, legumes, seeds, leaves, and shoots have been widely used as protein supplements and functional ingredients in the food industry [7,8,9]. Advancements in processing technologies further promote the applications of plant-based proteins in formulating alternative protein products.

The exploration of new food protein resources could expand the scope of protein acquisition, improve the efficacy of plant protein utilization, reduce dependence on animal-derived proteins, and contribute to environmental and food sustainability [10,11]. Notably, there has been a preliminary investigation into harvesting edible proteins from ginkgo seeds in China, driven by the increasing demand for plant proteins and the rising production of ginkgo seeds as a byproduct of the pharmaceutical industry [12].

*Ginkgo biloba* L., a tree species with a history dating back over 250 million years [13], has a widespread natural distribution and cultivation in many countries [14]. Highly valued for its ornamental, ecological, economic, and medicinal significance, ginkgo leaves, seeds, and extracts have attracted global interest [15,16,17]. Ginkgo seeds, renowned in China for thousands of years as dried fruits and traditional medicine [18], are recognized for various biofunctions such as immune regulatory, antioxidant, antibacterial, and anti-inflammatory effects [19,20]. Ginkgo seed protein (GSP), predominately composed of albumin and globulin, boasts high proportions of essential amino acids, making it a protein source with great developmental potential. However, like most plant storage proteins, GSP exhibits low solubility and poor gelling properties [21,22], hindering its utilization in the food sector. Consequently, there is a need to modify GSP to explore novel opportunities for developing functional protein ingredients from this natural source.

In addition to providing essential nutrients, food proteins contribute various functional properties to food products catering to diverse demands [23]. Gelation, an important functional property of proteins, plays a versatile role in shaping food texture and sensory properties. The gelling ability of proteins forms the foundation for various food forms and serves as a vehicle for delivering incorporated bioactive compounds [24]. Numerous approaches exist to modify the gelation properties of plant proteins, and simultaneous improvement of their techno- and bio-functional properties is particularly intriguing.

L-theanine (L-Th), a non-protein amino acid occurring naturally in the L configuration as N-ethyl-L-glutamine [25], is extracted from tea, particularly green, black, and oolong tea [26]. It exhibits various bioactivities, including anti-inflammatory, anti-tumor, antibacterial, antioxidant, anti-aging, immune regulatory, and stress-reducing properties [27,28]. Recognized for its health-promoting effects, L-Th has led to increased interest in developing related food additives and functional foods. Recent studies have also demonstrated that L-Th has food processing-enhancing effects. It can stabilize diacylglycerol emulsions and improve the antioxidant capacity of β-lactoglobulin [29]. In a dough system, L-Th promotes the formation of disulfide bonds and the beta-sheet structures of gluten, improving dough ductility [30]. A docking study suggests that L-Th can bind to the Lys residue of human serum albumin through hydrophobic interactions and hydrogen bonds [31], indicating a modifying effect on the protein structure. Given these findings, we hypothesized that incorporating L-Th into GSPI could improve its gelling properties and nutritional value. To the best of our knowledge, no studies have investigated the effect of L-Th on improving protein-based gels. Thus, the aim of this study was to investigate how the incorporation of different concentrations of L-Th affects the gelling properties of GSPI across different pH levels (5.0, 6.0, and 7.0).

## 2. Results and Discussion

### 2.1. Physicochemical Properties

#### 2.1.1. Solubility and Surface Hydrophobicity

**Figure 1:**
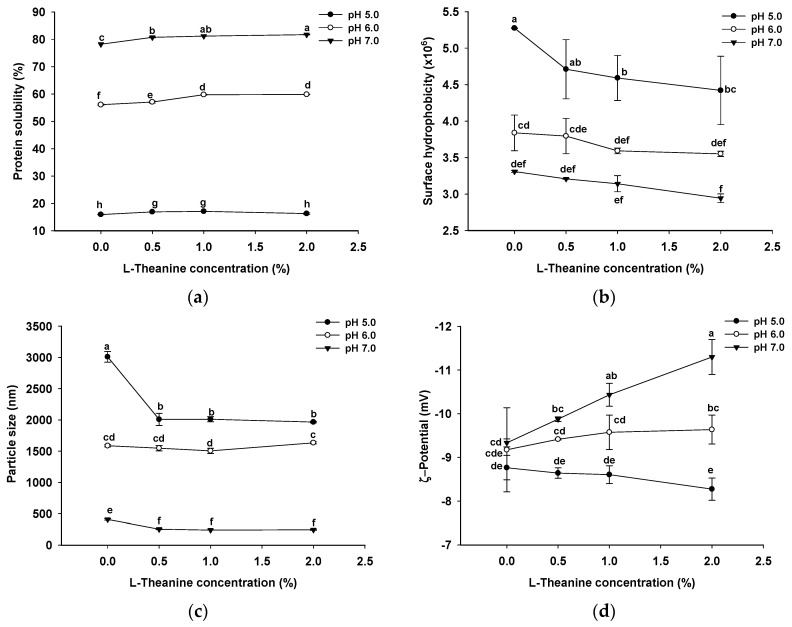
Physicochemical properties: protein solubility (**a**), surface hydrophobicity (**b**), particle size (**c**), and ζ potential (**d**) of GSPI sols at different pH levels and L-theanine concentrations. Values with different letters differ significantly (*p* < 0.05).

As shown in Figure 1a, the solubility was greatest at a pH of 7.0 (78.2 ± 0.0%), while it was lowest at a pH of 5.0 (15.9 ± 0.1%), given the proximity of the 5.0 pH level to the isoelectric point of GSPI (pI 4.4). The samples at a pH of 5.0 carried the fewest charges (−8.77 ± 0.28 mV), diminishing electrostatic repulsion and resulting in protein aggregation [32,33]. Conversely, at a pH of 7.0, farthest from the isoelectric point, increased electrostatic repulsion contributed to the highest solubility. With an elevation in the L-Th concentration at pH levels of 6.0 and 7.0, solubility gradually increased, while at a pH of 5.0, it initially increased and then decreased, possibly influenced by the protein particle size and electrostatic interactions. The results indicate that the addition of L-Th had little effect on GSPI solubility, whereas the pH level exerted a significant influence.

Surface hydrophobicity, a crucial property linked to protein conformations and flexibilities [34], serves as a relative index of solubility. The surface hydrophobicity of GSPI is shown in Figure 1b. The trend in the surface hydrophobicity of GSPI was opposite to that of solubility, with the highest surface hydrophobicity at a pH of 5.0 (5.27 × 10^6^ ± 4.45 × 10^3^), followed by pH levels of 6.0 (3.84 × 10^6^ ± 0.25 × 10^6^) and 7.0 (3.31 × 10^6^ ± 1.15 × 10^4^). At a pH of 5.0, weak electrostatic interactions due to a low charge led to predominantly hydrophobic protein--protein interactions, whereas at pH levels of 6.0 and 7.0, predominant protein--water interactions ensued due to proteins carrying accumulated negative charges [35]. Additionally, L-Th induced a decrease in surface hydrophobicity across all samples, though the changes were not statistically significant (*p* > 0.05).

#### 2.1.2. Particle Size and ζ Potential

In Figure 1c, the particle size of the control sol at a pH of 5.0 is the largest (3009 ± 86 nm) among the GSPI sols, which was likely a result of reduced electrostatic repulsion, leading to increased aggregate formation due to hydrophobic interactions. After the addition of L-Th, a decrease in particle size was observed at pH levels of 5.0 (33.1–34.7%) and 7.0 (37.7–40.9%), with a noticeable reduction at both pH levels as the L-Th concentrations increased. L-Th molecules, containing acetyl groups with an isoelectric point of 5.7, contributed to the observed changes [29]. However, the particle size increase in the sample with 2.0% L-Th at a pH of 6.0 might be attributed to the interplay of electrostatic repulsion and L-Th isoelectric point precipitation.

Figure 1d shows that at pH levels of 6.0 and 7.0, the ζ potential of the GSPI sol gradually increased in the presence of L-Th, while it decreased at a pH of 5.0. The isoelectric point of L-Th was 5.7, indicating that the changes were likely influenced by the negatively charged L-Th molecules at pH levels of 6.0 and 7.0 and positively charged L-Th molecules at a pH of 5.0. A high ζ potential typically leads to strong electrostatic stabilization of protein molecules, preventing their coalescence [36]. As the pH increased from 6.0 to 7.0, the L-Th with a low concentration did not increase the ζ potential of the GSPI, which is in agreement with previous findings [29].

### 2.2. Fluorescence Spectra

**Figure 2:**
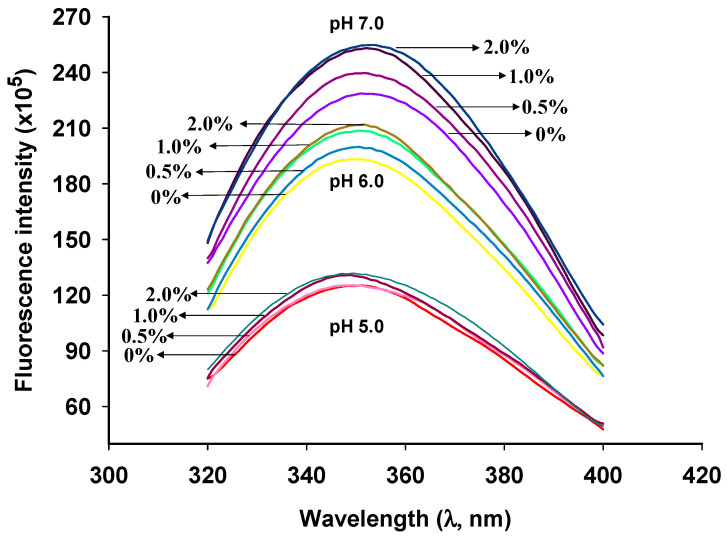
Intrinsic fluorescence spectroscopy of GSPI sols at different pH levels and L-theanine concentrations.

The tertiary structure of proteins can be analyzed using intrinsic fluorescence analysis. In this study, an excitation wavelength of 295 nm was used. At this wavelength, the endogenous fluorescence of the protein could be attributed solely to tryptophan (Trp) residues. Figure 2 shows the intrinsic fluorescence spectra of GSPI, revealing that the Trp fluorescence was highest for samples at a pH of 7.0 and lowest at a pH of 5.0. At a pH of 5.0, where surface hydrophobicity was elevated (Figure 1b), stronger protein–protein interactions may have led to steric hindrance, resulting in a decrease in fluorescence intensity [37]. Across all pH levels, an increase in GSPI fluorescence intensity was observed with higher L-Th concentrations. Particularly, the addition of L-Th significantly enhanced the GSPI fluorescence intensity with increasing pH levels. In the presence of L-Th, the high fluorescence intensity at pH levels of 6.0 and 7.0 could be attributed to reduced surface hydrophobicity and increased electrostatic repulsion, which led to protein unfolding [38]. In comparison with a pH of 5.0, the GSPI at pH levels of 6.0 and pH 7.0 showed a red shift. Typically, a low maximum emission wavelength (λ_max_) for Trp fluorescence indicates that Trp is buried in a non-polar environment [39]. The low λ_max_ at a pH of 5.0 may have been due to protein aggregation near the isoelectric point of GSPI, causing the Trp to move into a more hydrophobic environment [40]. Conversely, at pH levels of 6.0 and pH 7.0, the Trp gradually shifted toward the hydrophilic direction, resulting in a red shift [41]. 

### 2.3. Rheological Characteristics

**Figure 3:**
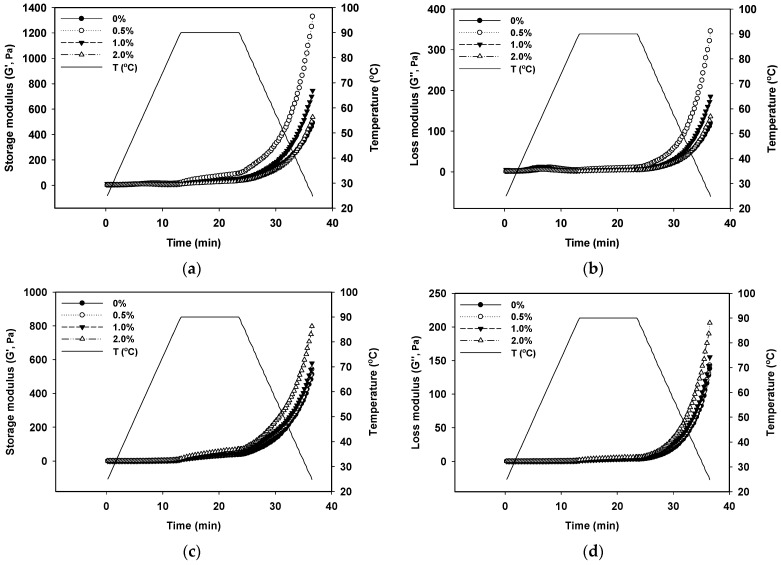

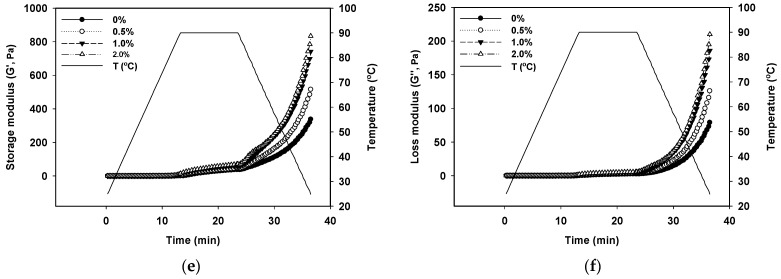
Storage modulus (G′) and loss modulus (G″) of GSPI gels at different pH levels ((**a**,**b**) pH 5.0, (**c**,**d**) pH 6.0, and (**e**,**f**) pH 7.0) and L-theanine concentrations.

A gel’s storage modulus (G′) and loss modulus (G″) are important indicators of its viscoelasticity. The G′ and G″ values of the samples are shown in Figure 3. The G′ and G″ of all samples barely changed below the denaturation temperature of GSPI (68.78 °C). G′ and G″ increased gradually when the temperature was held at 90 °C, and the increase became exponential during cooling. All gel samples displayed considerably higher G″ values than G′ values, indicating a predominately elastic behavior for the GSPI gels. This finding aligns with our previous observation [42]. Protein molecules began to unfold when the denaturation temperature was reached. This led to the exposure of nonpolar and sulfhydryl groups, facilitating the re-polymerization of proteins through hydrophobic interactions and intermolecular disulfide bonds and resulting in the formation of a three-dimensional gel network. This process contributed to the increase in both G′ and G″. In addition, the exponential rise in G′ and G″ observed during the cooling phase indicated a further strengthening of hydrogen bonds, reinforcing the gel structure [43,44,45]. At a concentration of 0.5% (*w*/*v*) for the L-Th, both G′ and G″ of the GSPI increased at a pH of 5.0. However, at L-Th concentrations of 1.0% and 2.0% (*w*/*v*), G′ and G″ decreased, possibly due to excessive L-Th aggregation near the isoelectric point, leading to precipitation. This in turn weakened the interaction with GSPI. Conversely, higher L-Th concentrations resulted in elevated G′ and G″ values at pH levels of 6.0 and pH 7.0. A plausible explanation is that more L-Th interacted with the GSPI, promoting the formation of hydrogen bonds and thereby strengthening the gel structure.

### 2.4. Analysis of Texture Profiles

**Figure 4:**
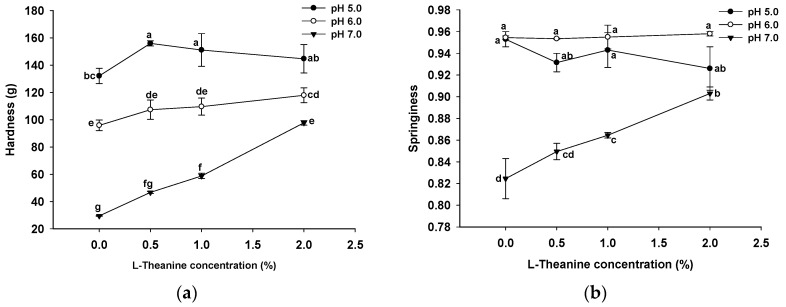

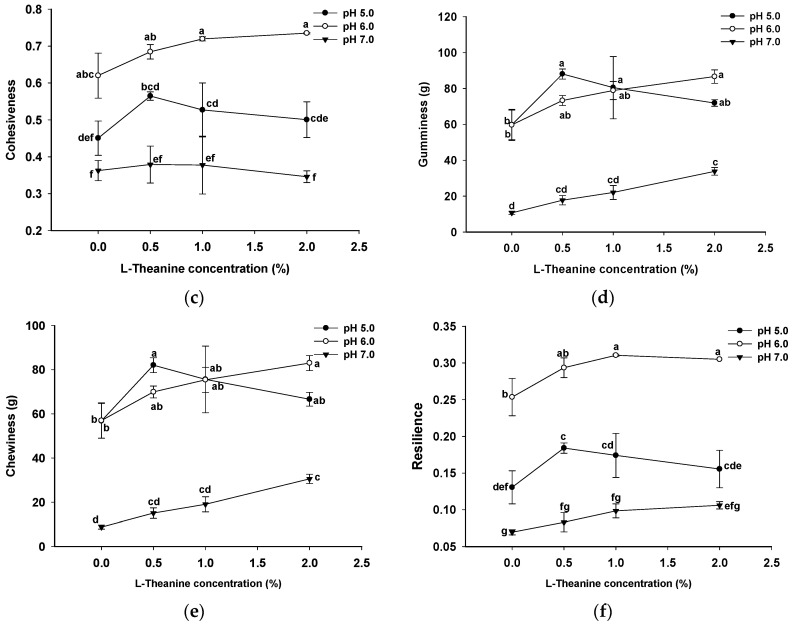
Texture profile analysis ((**a**) hardness, (**b**) springiness, (**c**) cohesiveness, (**d**) gumminess, (**e**) chewiness, and (**f**) resilience) of GSPI gels at different pH levels and L-theanine concentrations. Values with different letters differ significantly (*p* < 0.05).

The texture profile of GSPI gels at different pH values and L-Th concentrations is shown in Figure 4. The GSPI gels exhibited the highest hardness at a pH of 5.0 (132.1 ± 5.6 g), followed by pH levels of 6.0 (95.9 ± 3.9 g) and pH 7.0 (29.5 ± 0.2 g). At pH levels close to the isoelectric point of GSPI, weak electrostatic repulsion allows protein molecules to aggregate, resulting in loosely structured gels characterized by high hardness but poor springiness, cohesiveness, and resilience [46]. Conversely, when the pH level is far from the isoelectric point, electrostatic repulsion becomes the dominant force, leading to the formation of weaker gels [47]. The addition of L-Th slightly increased the hardness of the GSPI gels at pH levels of 5.0 (9.6–18.1%) and 6.0 (12.0–23.0%). In contrast, L-Th markedly increased the hardness (58.5–231.6%) and springiness (3.03–9.52%) of the gels at a pH of 7.0. The effect of L-Th on the cohesiveness of the GSPI gels was not significant at pH levels of 5.0 or pH 7.0, but it significantly improved the gel cohesiveness at a pH of 6.0. The gumminess, chewiness, and resilience of the gels followed a similar pattern to the hardness. L-Th may enhance the texture profiles of GSPI gels at pH levels of 6.0 and pH 7.0 by promoting intermolecular hydrogen bonding [38]. Increasing the surface charge of protein molecules enhances electrostatic repulsion between them, likely facilitating GSPI dispersion and the formation of fine gel structures, along with an increase in protein solubility (Figure 1a). At a pH of 5.0, increasing the L-Th concentrations resulted in a higher hardness, gumminess, and chewiness but lower resilience. This suggests that an excess of positively charged L-Th might weaken the electrostatic attraction between molecules.

### 2.5. Water Holding Capacity (WHC) and Gel Appearance

**Figure 5:**
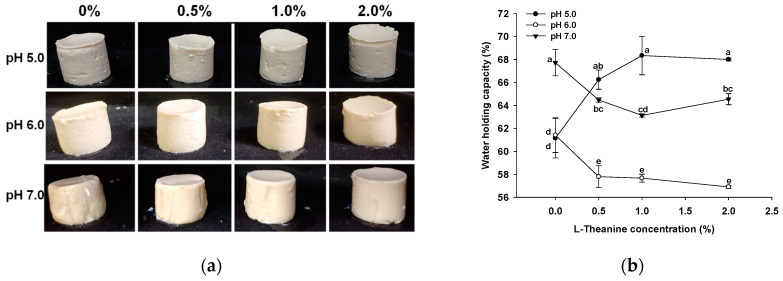
Appearance (**a**) and water holding capacity (**b**) of GSPI gels at different pH levels and L-theanine concentrations. Values with different letters differ significantly (*p* < 0.05).

Figure 5 provides a visual comparison of the appearances of the samples. At pH levels of 5.0 and 6.0, the L-Th did not significantly affect the GSPI gel appearance. However, at a pH of 7.0, the GSPI gels exhibited increased stiffness with increasing L-Th concentrations, aligning with the findings for the gel hardness (Figure 4a). In addition, the WHC of the GSPI gels, a critical aspect of a protein gel’s ability to retain water within its matrix, varied between 56.91% and 68.33% across different pH levels and L-Th concentrations (Figure 5b). At a pH of 7.0, the GSPI gels demonstrated a higher WHC compared with those at pH levels of 5.0 and 6.0. Near the protein isoelectric point, the formation of particulate-like and porous gels due to protein aggregation resulted in a relatively low WHC [48]. This trend is consistent with observations in whey protein gels [49]. At pH levels of 6.0 and 7.0, L-Th led to a slight increase in the WHC of the GSPI gels, whereas at a pH of 5.0, it had no effect.

### 2.6. Gel Microstructure

**Figure 6:**
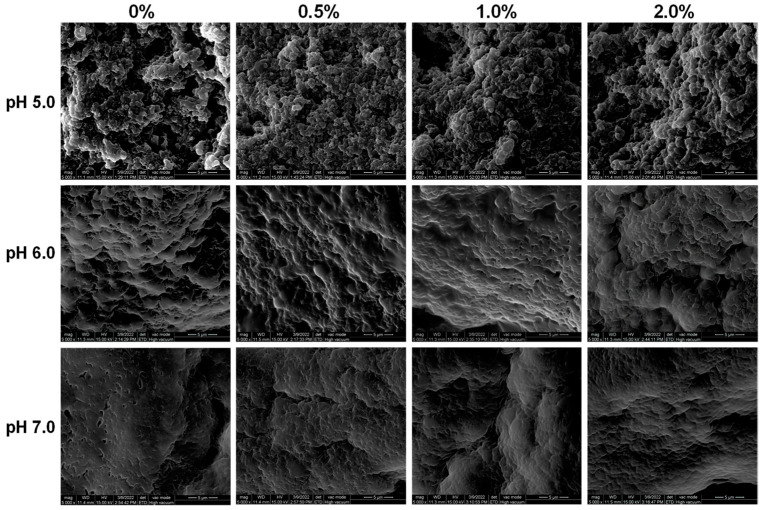
Scanning electron microscopy images of cross-section of GSPI gels at different pH levels and L-theanine concentrations.

SEM analysis was conducted to illustrate the microstructures of the GSPI gels. As shown in Figure 6, at a pH of 5.0, the GSPI gels were exhibited as aggregates of spherical protein particles, a phenomenon not observed at pH levels of 6.0 or 7.0. This aggregation might be attributed to weak electrostatic interactions near the protein’s isoelectric point. The addition of L-Th to the GSPI gels at a pH of 5.0 resulted in minimal changes in the network structure. In the pH 7.0 gels, the texture appeared homogeneous and dense, and the introduction of L-Th increased the electrostatic repulsion between the particles, leading to unfolding of the protein molecules. This elucidates how L-Th enhances the WHC in GSPI gels. Similarly, at a pH of 6.0, electrostatic repulsion reduced the graininess of the protein gels, facilitating water retention.

### 2.7. Fourier Transform Infrared Spectrum (FT-IR)

**Figure 7:**
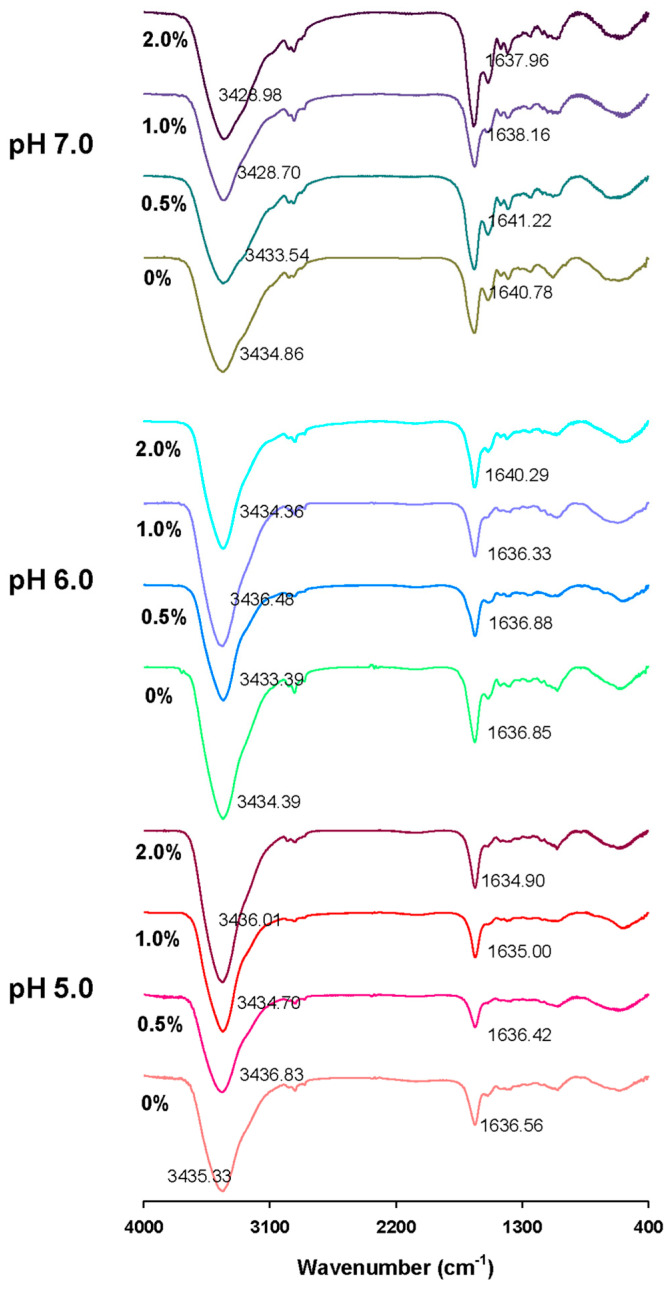
Fourier transform infrared spectrum of GSPI gels at different pH levels and L-theanine concentrations.

The FT-IR spectrum of GSPI gels at different pH levels and L-Th concentrations is shown in Figure 7. In the 3200–3600 cm^−1^ region (amide A), a broad absorption peak can be observed, attributed to the stretching vibration of intermolecular hydrogen-bonded N-H and O-H groups [50]. Changes in the secondary structure of the proteins can be analyzed in the 1600–1700 cm^−1^ region (amide I) and 1200–1350 cm^−1^ (amide III) region [51]. When examining the infrared spectrum of the GSPI gels, it is evident that L-Th had little effect on the protein’s primary structure. At a pH of 5.0, the peak of the L-Th-added samples did not exhibit apparent changes compared with the control group. A shift in the amide I wavenumber of the GSPI to a longer wavenumber at a pH of 6.0 suggests a further increase in the α-helical structure of the protein [52]. The interaction between L-Th and GSPI’s hydrogen bonds resulted in a shift in the peak of amide A to a shorter wavenumber at a pH of 7.0 [53]. The effects of L-Th on the structure of the GSPI vary under different pH conditions, and these effects become more pronounced when the protein is farther from its isoelectric point.

## 3. Conclusions

The strongest gels were achieved when the GSPI was close to its isoelectric point. The addition of L-Th to the GSPI gels did not improve their textural properties or WHC. However, the L-Th induced the formation of a fine GSPI gel structure and improved the gelling properties when the pH was far from the isoelectric point, leading to improved toughness and WHC values. The observed improvements in protein solubility, reduced particle sizes, and enhanced charge retention were likely attributed to the hydrogen bonding effects of L-Th. The impact of L-Th on the GSPI gels was influenced by both the pH and L-Th concentration. This is the first study to demonstrate the augmenting effect of L-Th on the functionalities of GSPI gels. L-Th could potentially play a dual role as a bioactive and techno-functional ingredient. It is worth noting that our study was confined to a relatively narrow pH range, and future investigations should expand to cover a broader spectrum of food and physiological conditions. Additionally, evaluating the stability and bioavailability of L-Th incorporated into GSPI gels will be crucial in assessing the potential of GSPI gels as a bio-delivery vehicle for L-Th.

## 4. Materials and Methods

### 4.1. Materials

*Ginkgo biloba* seeds were purchased from local markets (Xuzhou, Jiangsu, China). L-theanine (L-Th, >99% purity) was purchased from Huazhong Haiwei (Beijing, China) Gene Technology Co., Ltd. All other chemicals and reagents were purchased from Sigma Aldrich (St. Louis, MO, USA) and were of analytical grade.

### 4.2. Extraction of Ginkgo Seed Protein Isolate (GSPI)

Ginkgo seeds were deshelled, peeled, and dehydrated for 2 days at 40 °C. The dried ginkgo seed flour, sifted through an 80 mesh sieve, underwent defatting using n-hexane. The GSPI was extracted by the alkaline solubilization isoelectric point precipitation method [21]. Specifically, defatted ginkgo seed flour was dispersed in deionized water at a ratio of 1:10 (*w*/*v*) and adjusted to a pH of 9.0. After stirring for 30 min at room temperature (RT, 22 °C), the dispersion was centrifuged for 15 min at 10,000× *g*, 4 °C. The supernatant was collected, and the pH was adjusted to 4.4. The resulting supernatant was stirred at RT (22 °C) for 30 min and centrifuged as described above. The precipitate was collected, dispersed in deionized water, and neutralized. It was subsequently freeze-dried and kept in a refrigerator.

### 4.3. Preparation of the GSPI Sols and Gels

The sols and gels were prepared according to our previous study with minor modifications [42]. The GSPI was dispersed in deionized water to prepare a 24% (*w*/*v*) sol. After 6 h of stirring at RT, the sol was refrigerated overnight to ensure complete hydration. L-Th was dissolved in deionized water, stirred at RT until completely dissolved, and prepared in various concentrations (1.0%, 2.0%, and 4.0% *w*/*v*). The GSPI sol was mixed at a 1:1 ratio with water or different concentrations of L-Th solutions, yielding 6 mL of sols with a GSPI concentration of 12% (*w*/*v*) and L-Th concentrations of 0%, 0.5%, 1.0%, and 2.0% (*w*/*v*). These concentrations were chosen based on our previous studies to ensure proper gelation and observable changes in the gel properties. The pH of the mixed GSPI and L-Th sols was adjusted to 5.0, 6.0, and 7.0, representing the pH range of common food products. After stirring at RT for 1 h, all GSPI sols were heated in a 90 °C water bath for 30 min. Subsequently, the samples (GSPI gels) were cooled to RT and stored overnight in the refrigerator.

### 4.4. Solubility and Surface Hydrophobicity of the GSPI

Protein solubility was measured based on a previous study [54]. The samples were diluted to 2.0 mg/mL GSPI in 50 mM citrate-phosphate buffer at the appropriate pH levels (5.0, 6.0, and 7.0) and centrifuged at 5000× *g* for 10 min at 20 °C. The protein concentration of the obtained supernatant was determined using the biuret method. Solubility (%) was calculated based on the ratio of the supernatant protein concentration to the initial protein concentration before centrifugation.

Surface hydrophobicity was measured according to a previous study [21]. A GSPI sol (12%) was diluted to concentrations ranging from 0.1 to 0.5 mg/mL using 50 mM citrate-phosphate buffer. Anilino-1-naphthalenesulfonic acid (ANS) was added to the diluted solutions and left to react for 15 min in the dark. The fluorescence intensity was then measured using an excitation wavelength of 365 nm and an emission wavelength of 484 nm. The hydrophobicity index was calculated using the slope of the fluorescence intensity versus the protein concentration.

### 4.5. Particle Size and ζ Potential of the Sols

The particle size and ζ potential were determined using a Zetasizer Nano ZS90 instrument (Malvern Instruments Co., Ltd., Malvern, UK). To avoid multiple scattering effects, the GSPI sols were diluted to 2 mg/mL in 50 mM citrate-phosphate buffer of the appropriate pH before measurement [55].

### 4.6. Intrinsic Fluorescence

The GSPI sols were diluted to 1 mg/mL using 50 mM citrate-phosphate buffer at the appropriate pH for intrinsic fluorescence determination. The intrinsic fluorescence was measured with emission wavelengths between 320 and 400 nm and an excitation wavelength of 295 nm using a scanning speed of 1 nm/s [42].

### 4.7. Rheology

The rheological properties of the GSPI samples (2 mL) were measured using a DHR-1 rheometer equipped with a parallel plate 40 mm in diameter with a 1 mm gap. To prevent moisture loss and insulate heat, the samples were sealed with silicone oil. The temperature was ramped up from 25 °C to 90 °C at a rate of 5 °C/min, held at 90 °C for 10 min, and then rapidly cooled back to 25 °C at the same rate [56].

### 4.8. Texture Profile Analysis (TPA)

TPA of the gels was conducted using a TA-XT plus texture analyzer (Stable Micro Systems Ltd., Godalming, UK). The test was performed at a speed of 2 mm/s during the test, with the pre-test and post-test speeds set to 1 mm/s. A pressing distance of 7 mm and a trigger force of 5 g were used [22]. The hardness, springiness, gumminess, cohesiveness, resilience, and chewiness of the gels were evaluated.

### 4.9. Water Holding Capacity (WHC) of Gels

To measure the WHC, the gels were centrifuged at 3000× *g* for 20 min at 20 °C. Approximately 2 g of gels were placed in pre-weighed 5 mL centrifuge tubes (m_0_), and their weight was recorded as m_1_. Following centrifugation, excess water was removed with filter paper, and the weight was recorded as m_2_ [57]. The WHC was calculated using the following formula:

WHC (%) = (m_2_ − m_0_)/(m_1_ − m_0_) × 100%

### 4.10. Microstruture of the Gels

The gels were cut into small pieces, placed on bronze holders, and coated with gold. Subsequently, the microstructure of the gels was examined using a Quanta-200 scanning electron microscope (FEI Company, Eindhoven, The Netherlands) [58].

### 4.11. Fourier Transform Infrared Spectrum (FT-IR)

The FT-IT technique was conducted according to a previous study [59]. Approximately 1 mg of the lyophilized sample was ground with 100 mg of potassium bromide in an agate mortar. The entire process was carried out under infrared light. The resulting sample was placed into a mold, pressed into a tablet, and analyzed using an FT-IR spectrometer (VERTEX 80 V, Bruker, Germany). Measurements were made in the range from 4000 to 400 cm^−1^.

### 4.12. Statistical Analysis

Each measurement was conducted at least twice. Data were reported as mean values and standard deviations. Statistical analysis was performed using Statistix 9.0 (Analytical Software, Tallahassee, FL, USA). ANOVA followed by Fisher’s least significant difference test was used to detect statistical significance at α = 0.05.

## Data Availability

The data presented in this study are openly available in the article.
